# Suitability of spider mites and green peach aphids as prey for *Eriopis connexa* (Germar) (Coleoptera: Coccinellidae)

**DOI:** 10.1038/s41598-022-12078-8

**Published:** 2022-05-16

**Authors:** Sidnéia Terezinha Soares de Matos, Patrice Jacob Savi, Cirano Cruz Melville, Terezinha Monteiro dos Santos Cividanes, Francisco Jorge Cividanes, Daniel Júnior de Andrade

**Affiliations:** 1 College of Agricultural and Veterinary Sciences, São Paulo State University (UNESP), Via de Acesso Prof. Paulo Donato Castellane, s/n, Jaboticabal, São Paulo 14884-900 Brazil; 2 Laboratory of Plant Parasitology, Biological Institute of São Paulo, Ribeirão Preto, SP Brazil

**Keywords:** Agroecology, Zoology, Entomology

## Abstract

The ladybird *Eriopis connexa* is an important natural enemy of several pest arthropods in agroecosystems. High population of this predator is frequently observed in strawberry and soybean crops associated with spider mites. We used two-sex life table parameters to evaluate under laboratory conditions, the suitability of three species of spider mites (*Tetranychus evansi*, *Tetranychus urticae*, *Tetranychus ogmophallos*), and a species of aphid (*Myzus persicae*) as a prey for the predator *E. connexa*. *Eriopis connexa* completed immature development on all prey species except on *T. evansi*, in which all individuals of predator died before reaching the pupal stage. Among prey species that allowed the immature development of *E. connexa*, *T. urticae* and *M. persicae* provided a faster development time to the predator. Oviposition days, longevity and fecundity of *E. connexa* on *T. urticae* and *M. persicae* were substantially longer/higher than on *T. ogmophallos*. Net reproductive rate (*R*_0_), intrinsic rate of increase (*r*), and finite rate of increase (*λ*) of *E. connexa* feeding on *T. urticae* and *M. persicae* were also higher than those on *T. ogmophallos*. Based on the overall performance of the ladybird, the order of suitability of prey species was *M. persicae* > *T. urticae* > *T. ogmophallos* > *T. evansi*.

## Introduction

Spider mites and the green peach aphid *Myzus persicae* (Sulzer) (Hemiptera: Aphididae) are important pests in several crops, with the potential to cause significant yield losses^1,2^. The two‐spotted spider mite *Tetranychus urticae* Koch (Acari: Tetranychidae) is the most important pest mite worldwide for its extensive damage to several crops and numerous cases of pesticide resistance^3^. Τhe tomato red spider mite *Tetranychus evansi* Baker and Pritchard (Acari: Tetranychidae), and the peanut red spider mite *Tetranychus ogmophallos* Ferreira and Flechtmann (Acari: Tetranychidae) are emerging pests in tomato and peanut, respectively, and have caused considerable economic damage to these crops^4–6^.

Chemical control is the main method used for controlling populations of mites and aphids^6–8^. However, the efficacy of this method is not always effective, since these pests become more prone to evolving pesticide resistance due to their high biotic potential and high genetic variability^3,4,6,7^. Intensification of chemical sprays has led also to environmental pollution, and poisoning risks to farmers and consumers^8,9^. For this reason, alternative control measures have been sought worldwide^9–11^. Biological control is a potential alternative control method, mainly when combined with other control measures, thus helping to implement integrated pest management programs^11,12^.

Ladybirds (Coleoptera: Coccinellidae) have been used in farm systems as natural enemies of phytophagous arthropods and maintained some pests below the level of economically significant damage^12–14^. Among these ladybirds, *Eriopis connexa* (Germar) (Coleoptera: Coccinellidae) has a high pest-control potential due to its high foraging capacity, voracity, and polyphagy^14–17^. This ladybird is a Neotropical predator of several pest arthropods, including eggs of *Diuraphis noxia* (Mordvilko), *Rhopalosiphum maidis* (Fitch), *Rhopalosiphum padi* (L.), *Acyrthosiphon pisum* (Harris), *Schizaphis graminum* (Rondani), *Cinara atlantica* (Wilson) (Hemiptera: Aphididae), *Spodoptera frugiperda* (J.E. Smith) (Lepidoptera: Noctuidae)*, Diatraea saccharallis* (Fabricius) (Lepidoptera: Pyralidae), and *Macrosiphum euphorbiae* (Thomas) (Hemiptera: Aphididae)^12,15,17–20^.

*Eriopis connexa* is a control agent for aphids in pine, cotton, wheat, and citrus plants^13^. In Brazil, a field study of key mortality factors of *Myzus persicae* (Sulzer) (Homoptera: Aphididae) in cabbage recorded *E. connexa* as a potential control for this pest^21^. This predator was efficacious in controlling *Chaetosiphon fragaefolii* Cockerell (Hemiptera: Aphididae) on strawberry in a greenhouse in Argentina^22^. Because of its potential as an aphid predator, this ladybird was introduced into the United States to control the Russian wheat aphid, *D. noxia* and pea aphid *A. pisum*^20,23^. Preliminary investigations indicated that *E. connexa* is often associated with spider mites in strawberry and soybean crops (Matos and Andrade, personal information). However, studies that evaluated the suitability of spider mites for *E. connexa* performance are scarce. Knowing the suitability of a predator on different prey species can be useful for practical purposes in biological control programs^24,25^.

Life table parameters are accurate and reliable tools to track how prey species can be suitable for natural enemies given that such tools generate detailed information about the development, survival, longevity, fecundity, and life expectancy of a population^26^. These tools have been used to evaluate the suitability of several pests to various natural enemies^27,28^. To date, studies assessing suitability of prey species to *E. connexa* are limited only to some parameters such as immature development phase, longevity, and fecundity^13,16,18,29^. Furthermore, few studies that used life table parameters to evaluate suitability of prey species to this predator have been based on a female age-specific life table method^16,30,31^. In this method, only female individuals are taken into consideration disregarding the male population and variation in developmental rates among individuals of a population. According to Chi and Liu^32^, this may result to errors in the estimation of life table parameters. Given these limitations, Chi and Liu^26^ and Chi^32^ developed a theoretical model of life table analysis namely “two-sex life table”. That method considers ages or stages and development rates of both sexes. Such design allows deeper knowledge on biology of predators and population growth parameters, which are fundamental for pest management efficiency. Therefore, this study aimed to use the two-sex life table to evaluate the suitability of three species of spider mites (*T. evansi*, *T. urticae*, *T. ogmophallos*) and a species of aphid *M. persicae* as prey for *E. connexa*.

## Material and methods

### *Eriopis connexa* rearing

Rearing of *E. connexa* was initiated with specimens of adults collected from volunteer plants. Ladybirds were kept in cages made of a PVC tube (10-cm-height by 10-cm-diameter), lined internally with bond paper, and sealed with voile fabric. The insects were fed with eggs of *Ephestia kuehniella* Zeller (Lepidoptera: Pyralidae) and an artificial diet, which consisted of beer yeast and honey (1:1). Eggs laid by *E. connexa* were removed daily from the cages and kept in 12.0-cm diameter Petri dishes sealed with PVC film. The newly hatched larvae were transferred to new Petri dishes and fed with eggs of *E. kuehniella*. Water was supplied with a moistened polyethylene sponge^33^. The insects were maintained in a climate-controlled room at 25 ± 1 °C, 60 ± 10% relative humidity, and 12-h photoperiod.

### Plant materials

The seedlings of different plants used for the rearing of aphid and spider mites species in this study were purchased from "Casa de sementes" (Jaboticabal, São Paulo, Brazil). The plant materials used were obtained with prior permission, and the present study is in compliance with relevant guidelines and legislation.

### *Myzus persicae* rearing

Rearing of *M. persicae* started with insects collected from cabbage plants (*Brassica oleracea* L. var. *Acephala*) at São Paulo State University, Jaboticabal Campus. The rearing substrate was leaf discs (2.5 cm in diameter) from cabbage leaves with the abaxial side facing upward, which were placed in 5.0-cm-diameter Petri dishes filled with a 5.0-mm layer of 1% agar-water, for disc turgidity. Nymphs and adults of *M. persicae* were placed on the discs, and the Petri dishes were sealed with PVC film and kept in a climate-controlled chamber at 23 ± 1 °C, 70 ± 10% RH, and 12-h photoperiod. The agar-water layer and leaf discs were changed twice a week.

### Rearing of spider mites

The original colonies of *T. urticae*, *T. evansi*, and *T. ogmophallos* used in this study was established from jack bean (*Canavalia ensiformis* L. cv. commun), tomato (*Solanum lycopersicum* L. var. cerasiforme), and peanut (*Arachis hypogaea* L., cv. Granoleico) plants, respectively, maintained in a screen house of the Acarology laboratory at São Paulo State University, Jaboticabal Campus, Brazil. After identification of the spider mite species, live specimens from each species were used to establish the laboratory colony on the same plant species as in the original colony. The host plants were 35–60 day-old and were grown into 5-L pots which were by 80% filled with a homogeneous mixture of soil, sand, and tanned bovine(1:1:1). All spider mite specie were kept climate-controlled rooms at 25 ± 1 °C, 60 ± 10% relative humidity, and 12-h photoperiod. Plants deteriorated by mites and/or senescent were periodically replaced with new ones.

### Experimental procedure

**Figure 1:**
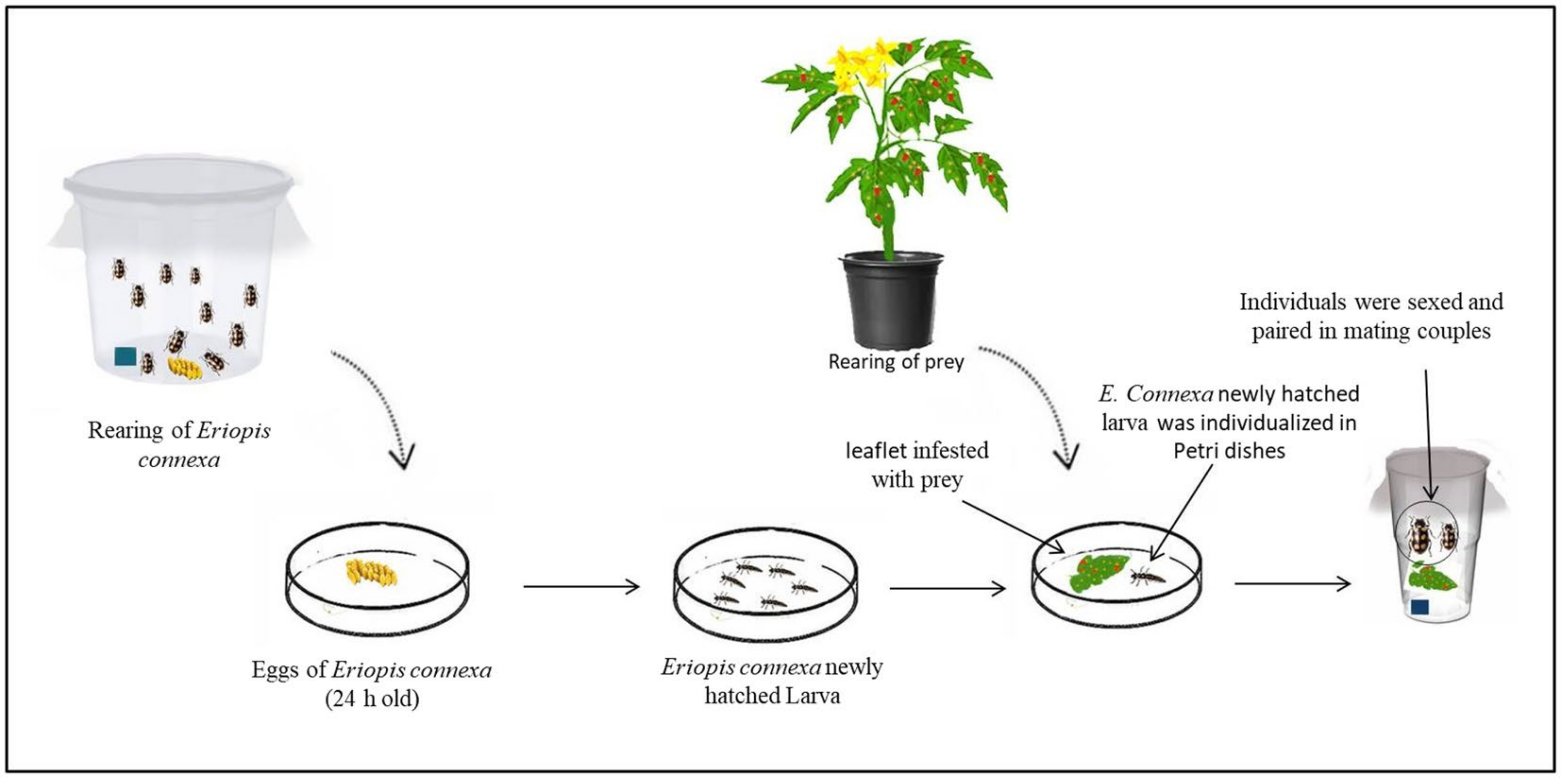
Experimental set up of *Eriopis connexa* reared on *Tetranychus evansi*. The same experimental set up was followed for the experiments of *Eriopis connexa* on *Tetranychus urticae*, *Tetranychus ogmophallos* and *M. persicae.* Number of replications = 50.

Development and reproduction of *E. connexa* were evaluated in four groups of prey diet: *T. urticae*, *T. evansi*, *T. ogmophallos*, and *M. persicae* under the same conditions described above for the stock colony. Initially, 10 pairs of freshly emerged females and males of *E. connexa* were maintained in a 350-mL transparent plastic cup (7 cm in diameter and 10 cm in height) covered by fine nylon netting (40 mesh), to mate and lay eggs. Fifty freshly deposited eggs (< 1-day old) were transferred into 12.0-cm-diameter Petri dishes sealed with PVC film until hatching (Fig. 1). Newly hatched larvae (< 24 h old) were placed individually in 9-cm-diameter Petri dishes, sealed with PVC film. A cohort of 50 larvae of *E. connexa* was used per prey diet (number of replications). A surplus of 200 individuals with the same ratio of different stages of prey was provided to each predator stage daily to ensure an abundance of food. Upon reaching adulthood, ladybirds were sexed and transferred in couples to new plastic cups. In the case of treatments where the number of females emerged was greater than the number of males, some males from the rearing stock were used to form couples (Fig. 1). As a source of water, a small Petri dish with wetted cotton wool (3 cm in diameter) was used. The experimental units were examined every 24 h to determine the duration of each developmental stage and survivorship. The pre-oviposition period (APOP: period between adult emergence and its first oviposition), total pre-oviposition period (TPOP: period from egg to first oviposition), oviposition days (number of days in which oviposition occurred), longevity of each sex, sex ratio and fecundity were also determined. In units where males died before females, other males from the stock colony were used to replace them. Data on males that came from stock colonies to form the couples or those used to replace died males before females were not used in statistical analysis. Eggs laid were removed at each observation time. The experiment was considered complete after all predators had died.

### Complementary test

As *E. connexa* was unable to complete its immature development phase feeding only on *T. evansi*, a complementary test was performed to evaluate its development when fed with *T. evansi* combined with a complementary food source. For this purpose, ladybird larvae were daily fed only with *T. evansi* until the fourth instar, and after that, *T. urticae* were added to the diet. Larval mortality and adult emergence were recorded.

### Statistical analyses

The software TWOSEX-MSChart by Chi^34^ available at http://140.120.197.173/Ecology/prod02.htm was used to estimate development and reproduction raw data and to calculate population parameters, using the procedure “two-sex life table”^26,32^. The following parameters were estimated: age-stage–specific survival rate (*s*_*xj*_), age-specific survival rate (*l*_*x*_), age-specific fecundity (*m*_*x*_), net reproduction rate (*R*_0_), intrinsic rate of increase (*r*), stage-specific fecundity (*f*_*xj*_), finite rate of increase (*λ*), average generation time (*T*), age-stage life expectancy (*e*_*xj*_), and age-stage reproductive value (*v*_*xj*_).

The variance and standard errors of development, fecundity, reproduction period, population parameters and survival curves were estimated using the bootstrap^35–38^. During this procedure, data of each of these biological parameters were re-sampled 100,000 times. Differences among treatments were compared by the paired bootstrap test, based on the 95% confidence interval of differences implemented in the TWOSEX-MSChart^39,40^.

## Results

### Life stage duration, fecundity, sex ratio, and life table parameters

The duration of the larval stages of the *E. connexa* was substantiality shorter on *M. persicae* and *T. urticae* than on *T. ogmophallos, and T. evansi,* except for the first instar of the last prey species (Table 1). Similar pattern was observed for the predator pupal stage, also shorter on the former two prey species (Table 1)*.* Thus, the immature development time for both sexes was shorter on *M. persicae* (18.4 ± 0.28 and 18.3 ± 0.31 days, respectively for male and female) and *T. urticae* (18.7 ± 0.27 for both sexes) than on *T. ogmophallos* (22.7 ± 0.24 and 22.8 ± 0.24 days, respectively for male and female). The immature survival of *E. connexa* fed with *M. persicae* and *T. urticae* was higher than 95% (Table 1). Sex ratio was significantly higher on the latter previously mentioned prey species than *T. ogmophallos. Eriopis connexa* fed with *T. evansi* could not complete its developmental cycle, with no larva reaching the pupal stage (Table 1). However, in the complementary test, *E. connexa* larvae fed with *T. evansi* completed their biological cycle after *T. urticae* was added to the diet, at the beginning of the fourth instar (Table 2).

**Table 1:** Means (± SE) of duration (days) of immature stages of *Eriopis connexa* fed on *Tetranychus urticae*, *Tetranychus evansi, Tetranychus ogmophallos*, or *Myzus persicae* at 25 ± 1 °C, 70 ± 10% RH, and 12-h photoperiod. Numbers in parentheses: number of surviving specimens at each stage.

Stage	*Tetranychus urticae*	*Tetranychus evansi*	*Tetranychus ogmophallos*	*Myzus persicae*
Larva (1st instar)	2.2 ± 0.05 b (50)	2.3 ± 0.0 b	3.1 ± 0.07 (50) a	2.2 ± 0.06 (48) b
Larva (2nd instar)	2.3 ± 0.06 b (50)	3.1 ± 0.1 a	2.9 ± 0.08 (37) a	2.3 ± 0.06 (48) b
Larva (3rd instar)	2.5 ± 0.07 b (50)	3.4 ± 0.1 a	3.4 ± 0.08 (37) a	2.5 ± 0.07 (48) b
Larva (4th instar)	4.3 ± 0.07 b (50)	–	4.6 ± 0.08 (37) a	3.9 ± 0.1 (48) c
Pre-pupa	1.0 ± 0.0 a (50)	–	1.0 ± 0.0 (37) a	1.0 ± 0.0 (48) a
Pupa	3.4 ± 0.07 b (48)	–	4.3 ± 0.08 (37) a	3.5 ± 0.07 (48) b
Larva-adult	15.8 ± 0.07 b (48)	–	19.5 ± 0.07 (37) a	15.4 ± 0.07 (48) b
Egg (second offspring)	3.0 ± 0.0 b (50)	–	3.4 ± 0.10 (50) a	3.0 ± 0.0 (50) b
Immature stage duration				
Female	18.7 ± 0.19 (25) b	–	22.7 ± 0.24 (17) a	18.4 ± 0.28 (25) b
Male	18.7 ± 0.27 (23) b	–	22.8 ± 0.24 (20) a	18.3 ± 0.31 (23) b
Survival rate	0.97 ± 0.0 (48) a	–	0.73 ± 0.1 (37) b	0.97 ± 0.0 (48) a
Sex ratio [(♀/(♂ + ♀)]	0.52 ± 0.04 a	–	0.46 ± 0.03 b	0.52 ± 0.04 a

Means followed by the same letters in a row do not differ from each other. Standard errors (SE) were estimated using 100,000 bootstrap resamplings and means were compared by paired bootstrap test (B = 100,000) based on 95% confidence interval of the difference between.

**Table 2:** Means (± SE) of duration (days) of immature stages of *Eriopis connexa* fed on *Tetranychus evansi* until the fourth instar, and with supplementary prey of *Tetranychus urticae* after the fourth instar*,* at 25 ± 1 °C, 70 ± 10% RH, and 12-h photoperiod. Numbers in parentheses: number of surviving specimens at each stage.

Stages	Larva				Pre-pupa	Pupa	Larva-adult
	(1st instar)	(2nd instar)	(3rd instar)	(4th instar)			
	2.3 ± 0.0(20)	3.1 ± 0.1(20)	3.4 ± 0.1(18)	4.2 ± 0.08(18)	1.0 ± 0.0(18)	3.8 ± 0.08(18)	17.8 ± 0.2(18)

Pre-oviposition and total pre-oviposition periods were significantly shorter for *E. connexa* fed with *M. persicae* and *T. urticae* compared to those fed *T. ogmophallos* (Table 3). Likewise, longevity, fecundity, and oviposition days increased for the predator when with fed *T. urticae* and *M. persicae* (Table 3). All parameters for the predator population growth were significantly influenced by the prey species (Table 3). Net reproduction rate (*R*_0_), intrinsic rate of increase (*r*), and finite rate of increase (*λ*) were significantly higher for ladybirds fed with *M. persicae* and *T. urticae* compared to those fed with *T. ogmophallos*.

**Table 3:** Means (± SE) of duration (days) of pre-oviposition period (APOP), total pre-oviposition (TPOP), longevity, oviposition days, fecundity and life-table parameters of *Eriopis connexa* fed on *Tetranychus urticae*, *Tetranychus ogmophallos*, or *Myzus persicae* at 25 ± 1 °C, 70 ± 10% RH, and 12-h photoperiod.

Parameter	*Tetranychus urticae*	*Tetranychus ogmophallos*	*Myzus persicae*
APOP (days)	7.9 ± 0.29 (25) b	12.0 ± 0.72 (17) a	7.1 ± 0.24 (25) b
TPOP (days)	26.6 ± 0.36 (25) b	34.7 ± 0.71 (17) a	25.5 ± 0.35 (25) b
Female longevity (days)	93.9 ± 2.5 (25) a	27.94 ± 2.44 (17) b	93.5 ± 1.91 (25) a
Male longevity (days)	90.0 ± 2.2 (23) a	26.7 ± 2.2 (20) b	93.2 ± 2.31 (23) a
Total longevity (days)	107.1 ± 3.0 (50) a	38.7 ± 3.0 (50) b	107.4 ± 3.35 (50) a
Oviposition days	35.4 ± 0.78 (25) a	3.5 ± 0.32 (17) b	34.8 ± 1.18 (25) a
Fecundity (eggs/female)	452.2 ± 12.2 (25) a	19.3 ± 1.9 (17) b	467.6 ± 19.35 (25) a
*R*_0_ (eggs/individual)	226.11 ± 32.51 (50) a	6.56 ± 1.43 (50) b	233.82 ± 34.46 (50) a
*r* (day^−1^)	0.118 ± 0.004 (50) a	0.047 ± 0.005 (50) b	0.126 ± 0.005 (50) a
*λ* (day^−1^)	1.125 ± 0.005 (50) a	1.048 ± 0.006 (50) b	1.133 ± 0.006 (50) a
*T* (day)	45.96 ± 0.801 (50) a	40.17 ± 1.07 (50) b	43.41 ± 06.9 (50) ab

Numbers in parentheses: number of individuals for a respective parameter. Means followed by the same letters in a row do not differ from each other. Standard errors (SE) were estimated using 100,000 bootstrap resamplings and means were compared by paired bootstrap test (B = 100,000) based on 95% confidence interval of the difference between. Numbers in parentheses: number of surviving specimens at each stage.

### Age- and stage-specific survival and fecundity rate

**Figure 2:**
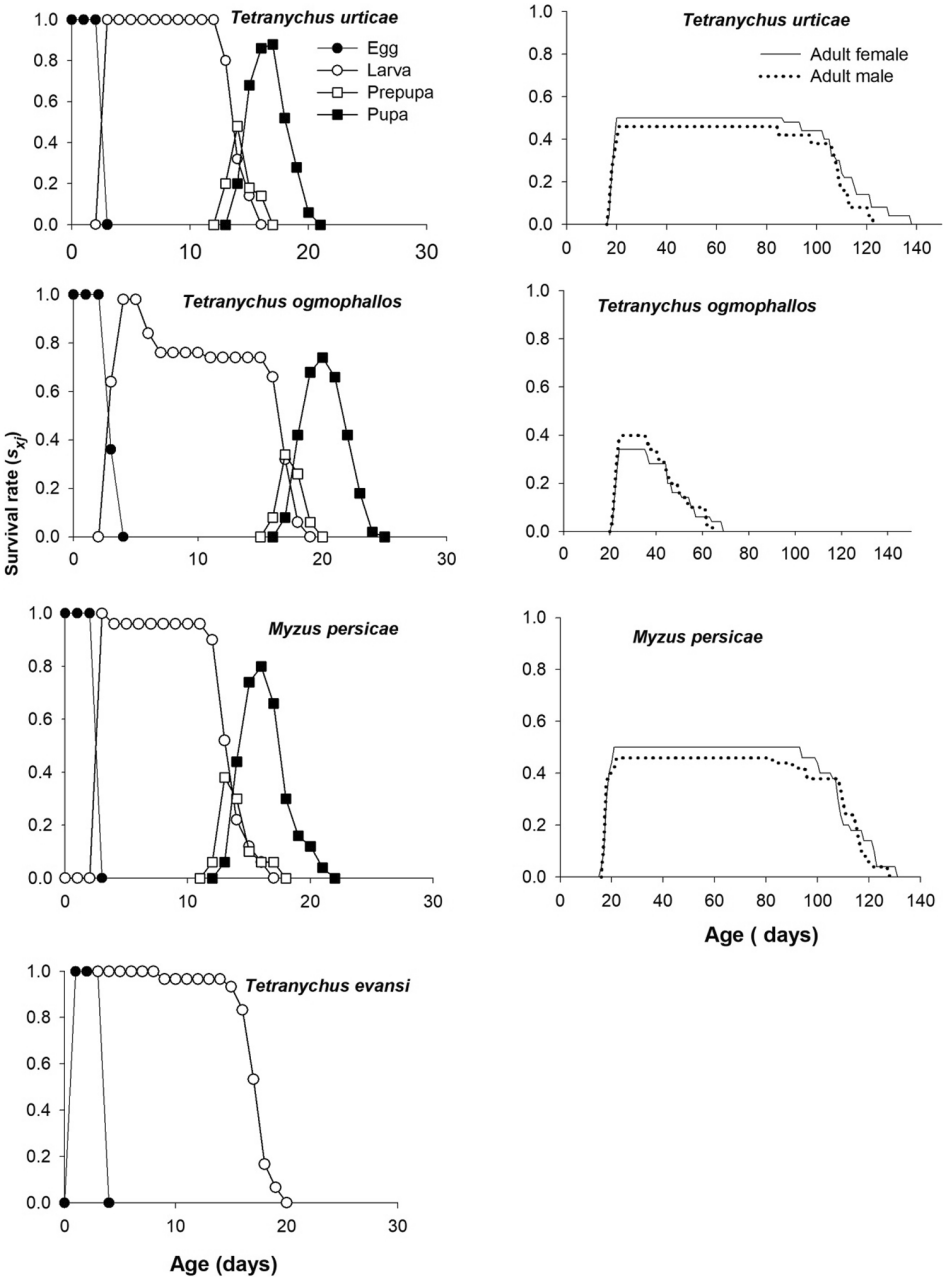
Age-stage-specific survival rate (*s*_*xj*_*)* of *Eriopis connexa* fed on *Tetranychus urticae*, *Tetranychus evansi, Tetranuchus ogmophallos*, or *Myzus persicae*.

Survival-rate curves (*s*_*xj*_) show the probability of a freshly oviposited egg to survive to age *x* and develop to stage *j* (Fig. 2). Due to changes in the development and survival, the stages of the predator fed with different prey, the *s*_*xj*_ curves showed significant overlap. The probability of a freshly laid egg surviving to the adult stage was higher for ladybird adults fed with *T. urticae* and *M. persicae* (0.5 for females and 0.46 for males) than for those fed with *T. ogmophallos* (0.34 for females and 0.40 for males). The probability of newly hatched larvae of the predator reaching the adult stage of when feeding on *T. evansi* was zero (Fig. 2).

**Figure 3:**
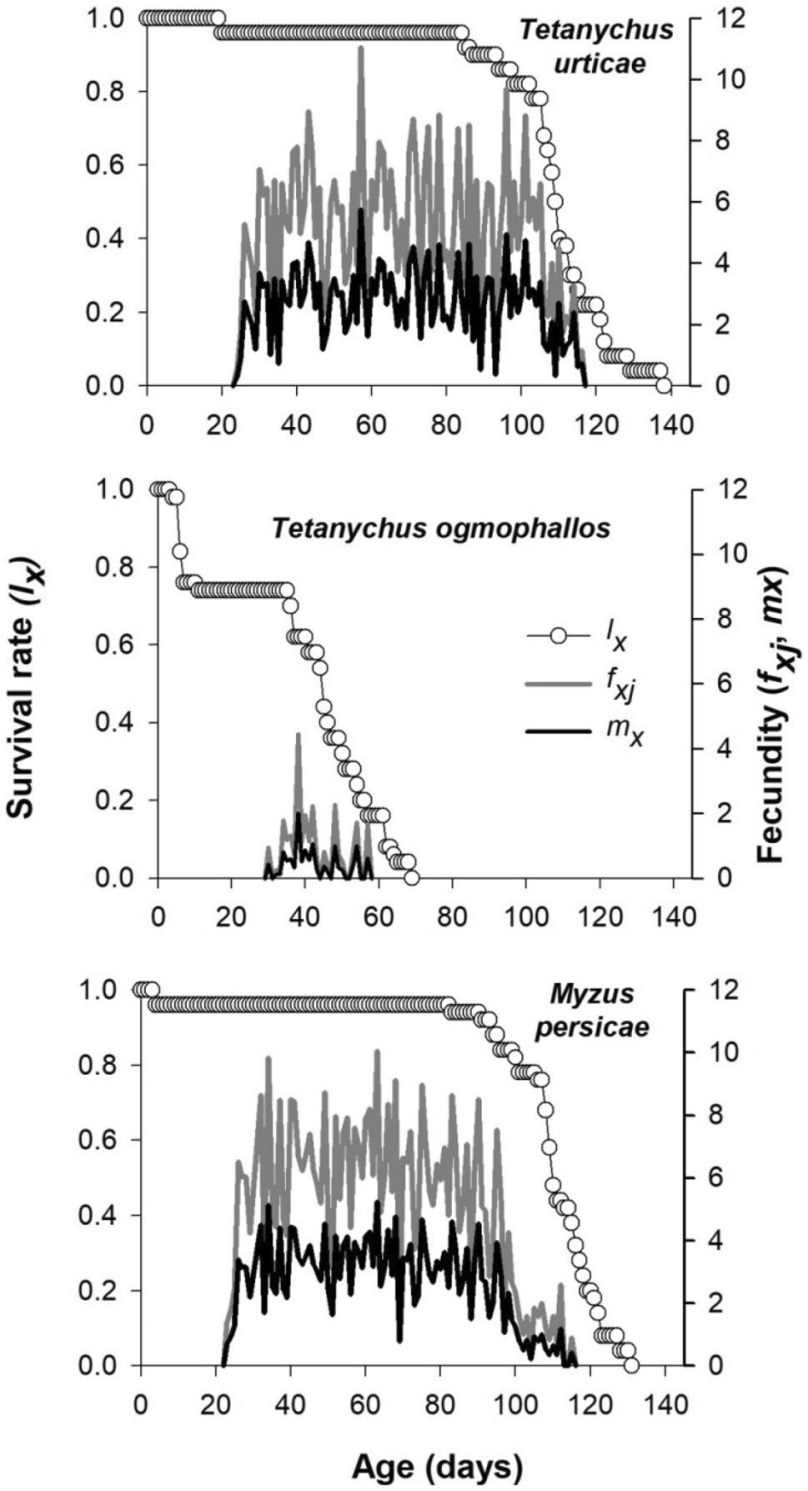
Age-specific survival rate (*l*_*x*_), age-specific fecundity (*m*_*x*_), and age-stage-specific fecundity (*f*_*xj*_) of *Eriopis connexa* fed on *Tetranychus urticae*, *Tetranychus ogmophallos*, or *Myzus persicae*.

Age-specific survival rate (*l*_*x*_) expresses the survival probability that an individual of *E. connexa* would survive until age x (Fig. 3). The 84% of *E. connexa* individuals fed with *M. persicae* and *T. urticae* remained alive for 96.0 ± 5.14 days and 98.0 ± 6.03 days, respectively, showing a significantly higher *l*_*x*_ than that of *T. ogmophallos* (6.0 ± 3.3 days) (Fig. 3). Similar patterns were observed for 50% and 16% of ladybirds’ survival on *M. persicae* (110.0 ± 1.79 and 122.0 ± 2.06 days, respectively) and *T. urticae* (109 ± 1.06 and 122.0 ± 2.85 days, respectively) which were higher than on *T. ogmophallos* (45.0 ± 2.37 and 57.0 ± 3.48 days, respectively). Age-stage-specific fecundity *f*_*x4*_ **(**daily number of eggs produced per female of age x) showed higher egg-laying peaks for females of *E. connexa* fed with *T. urticae* (11.04 eggs) and *M. persicae* (10.03 eggs) on the 58th and 63rd day of their age, respectively. For female adults fed with *T. ogmophallos*, the egg-laying peak (4.4 eggs) occurred at 38 days of age (Fig. 3).

**Figure 4:**
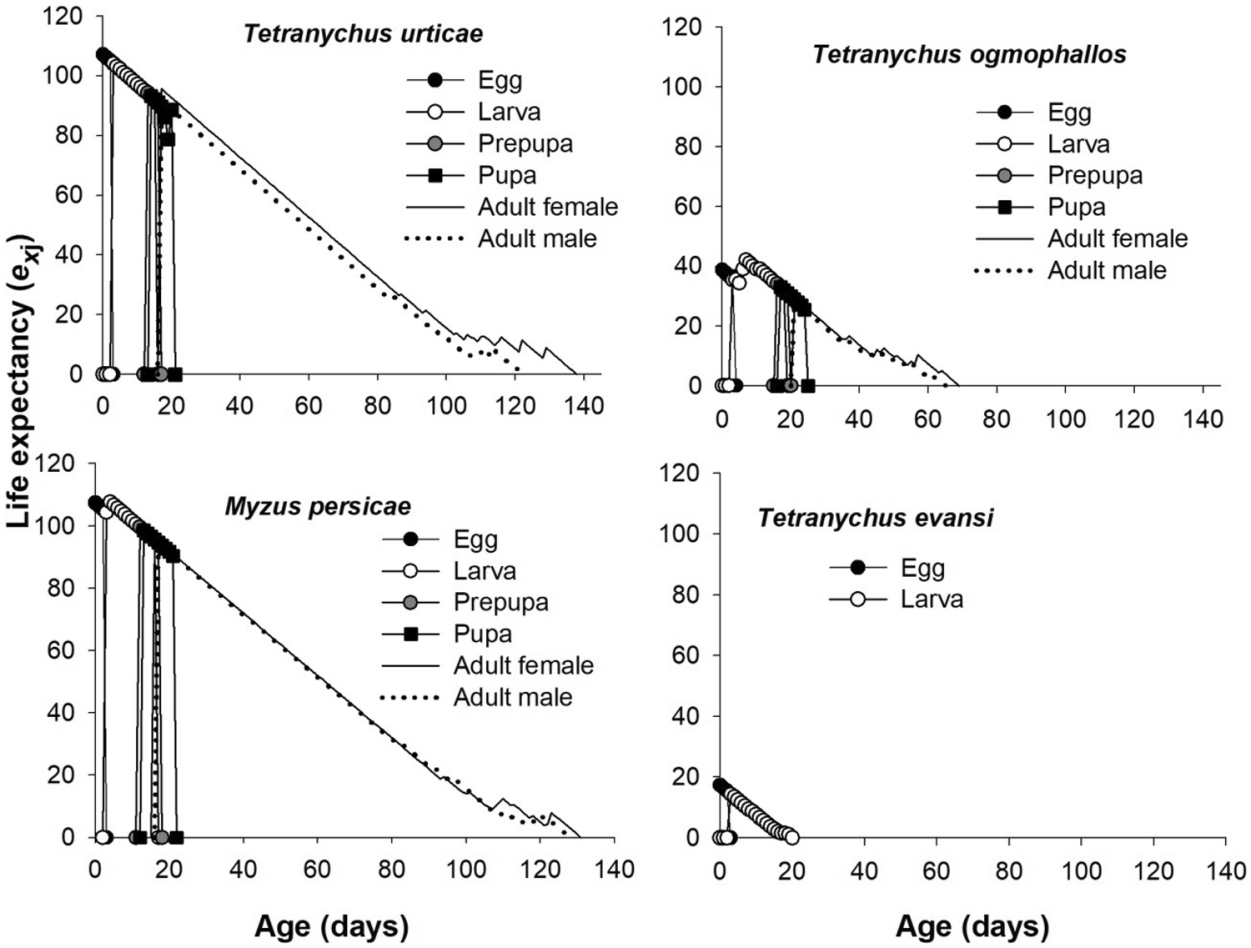
Age-stage life expectancy (*e*_*xj*_) of *Eriopis connexa* fed on *Tetranychus urticae*, *Tetranychus evansi, Tetranychus ogmophallos*, or *Myzus persicae*.

Age-specific fecundity *m*_*x*_ (average daily fecundity per individuals at age x) showed the highest value when *E. connexa* fed with *T. urticae* (5.83 eggs) and *M. persicae* (5.13 eggs) at 57 and 49 days of age, respectively (Fig. 3). The life expectancy of freshly laid eggs (*e*_*01*_) was higher (about 107 days) for *E. connexa* fed with *T. urticae* and *M. persicae* than for those fed with *T. ogmophallos* (38.7 days) (Fig. 4). Therefore, adult females at 60 days of age can still live about 52 days feeding on these prey species. In contrast, the life expectancy was 18 and 39 days in adult ladybirds fed with *T. evansi* and *T. ogmophallos*, respectively.

**Figure 5:**
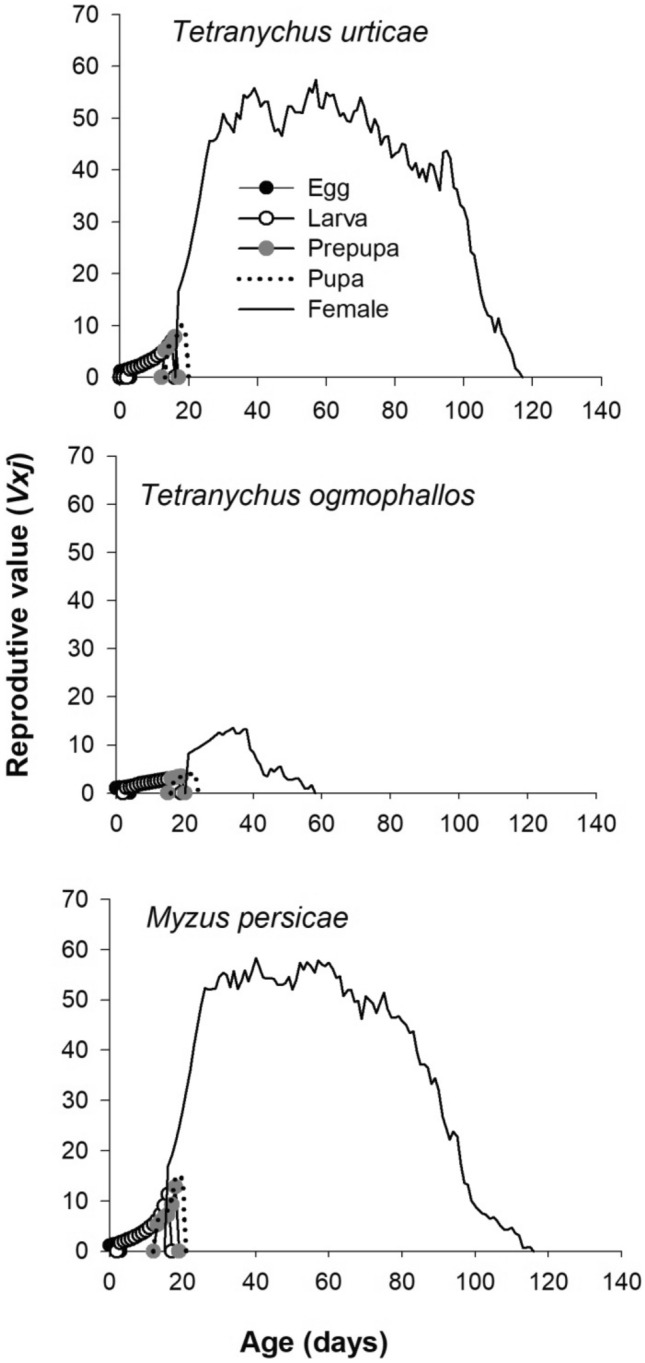
Age-stage reproductive value (v_xj_) of *Eriopis connexa* fed on *Tetranychus urticae*, *Tetranychus ogmophallos*, or *Myzus persicae*.

Age-stage reproductive value (*v*_*xj*_) of *E. connexa* adult females contributed more to population growth than other development stages. The peaks (*v*_*xj*_) of adult females fed with *M. persicae, T. urticae*, or *T. ogmophallos* were 58.4, 55.8, and 13.5, respectively (Fig. 5).

## Discussion

Prey quality is a key factor affecting the growth, development and reproduction of predatory insects^41^. The suitability of a prey species can be evaluated by measuring its effect on biological attributes of the predator^42^. Prey are categorized as essential (supports development and reproduction), alternative (supports only survival) or rejected on the basis of quantitative data on the developmental rate, survival and reproductive capacity^43–45^. Due to the importance of predatory ladybirds in biological control, this study investigated effects of three spider mite species (*T. evansi*, *T. urticae*, and *T. ogmophallos*) and an aphid species (*M. persicae*) on the development, survival and reproductive performance of the predator *E. connexa*. Our findings indicated that *E. connexa* was able to complete its life cycle and reproduce on all prey studied, except for *T. evansi,* a diet that resulted in 100% mortality of the predator before reaching the pupal stage. However, the prey species that allowed the development of *E. connexa* had a strong influence on demographic parameters of the predator. For instance, *E. connexa* fed with *T. urticae* and *M. persicae* showed a faster immature developmental period and high survival rate when compared to the *T. ogmophallos* diet. Furthermore, the oviposition time, longevity for both sexes and fecundity on *T. urticae* and *M. persicae* diets were longer/higher when compared to *T. ogmophallos.* These results suggest that *T. urticae* and *M. persicae* are more suitable prey species for *E. connexa*, probably due to the quality difference in nutrient contents of the prey species^46–48^. Several studies reported that the variation of the chemical profiles, morphological or allelochemical features of host plants have also a direct effect on the nutritional value of herbivorous arthropods, in terms of their suitability for predators^41,49,50^. In our study, the prey species have been reared on different host plant families (*B. oleracea*, *C.ensiformis, A.hypogaea cv. Granoleico* and *S. lycopersicum*) that will presumably tend to have higher differences. Therefore, although not being evaluated in the present study, this fact could also have contributed as an additional cause of difference in the quality of prey species that affected development, survival and reproductive performance of *E. connexa*.

Most of the results obtained in this study are comparable to those of previously studies, which report that the type of prey had a significant influence on the developmental period, survival rate, and reproduction of several other ladybirds^47,51,52^. For instance, the duration of *E. connexa* immature phase (larva-adult) reported by Silva et al.^16^ on five prey species [immature of *E. kuehniella, S. frugiperda*, *D. saccharalis*, *R. maidis*, *S. graminum*] at 25 or 26 °C, lasted 12.6–17.4 days, values similar to those found in our study. Values of *E. connexa* development time (larva-adult) found by Nascimento et al.^16^ on *Plutella xylostella* (L.) larvae, the brassica aphid, *Lipaphis pseudobrassicae* (Davis) when provided separate or mixed are also comparable (17.1–24.7 days) to those of this study. On the other hand, Duarte et al.^30^ reported higher developmental duration (22.9–47.48 days) on three aphid species [*Brevicoryne brassicae* L., *Macrosiphum euphorbiae* Thomas, and *Pterocallis* sp. (Hemiptera: Aphididae)], but similar survival rates (37.5–97.5%) with our findings.

Sarmento et al.^53^ have reported an exponential (Type I) functional response for adults of *E. connexa* fed on *T. evansi*, but the authors did not assess the subsequent effects on different developmental stages and reproduction resulting from that predator feeding with *T. evansi* diet, an aspect fundamental in conservation and growth of the predator in agro-system. In this study, we found that *T. evansi* is unsuitable for the development and reproduction of *E. connexa.* Similar results have been reported by Oliveira et al.^54^ for *Cycloneda sanguinea* (Linnaeus) (Coleoptrea: Coccinellidae) which did not complete successfully its biological cycle feeding on *T. evansi*. Indeed, the mite is known as prey that sequesters toxic secondary metabolites from plants and accumulates them in its body^55^, which possibly makes it unsuitable as a prey for *E. connexa*. This is also supported by the increased mortality during the last instar, since ladybird larvae need more nutrients to reach the pupal and adult stage^56^, i.e., food consumption and intake of toxic compounds increased during L4^57^. Furthermore, when the diet changed to *T. urticae* at the beginning of the fourth instar, the predator successfully completed the immature development phase. This result also indicates the toxic effect of *T. evansi* on *E. connexa* development can be reversible in the presence of other diet. Likewise, Munyaneza and Obrycki^58^ demonstrated that *Leptinotarsa decemlineata* (Say) (Coleoptera: Chrysomelidae) eggs are an adequate diet for *Coleomegilla maculata* DeGeer (Coleoptera: Coccinellidae) only when the larva consumes aphids in the early stages of development. Nascimento et al.^16^ also found that the performance (developmental time and survival) of *E. connexa* improved when larvae fed on diets of mixed prey compared to simple *P. xylostella* larvae prey diet*.* However, the effects of mixed diets that include *T. evansi* need to be assessed in further studies to investigate this relationship.

Zazycki,et al.^31^ reported longevity and fecundity of *E. connexa* on pollen + *E. kuehniella* eggs at 25 °C (60 –130 days; 584 ± 96.50 eggs/female respectively), which were comparable to those found on *T. urticae* and *M. persicae* in this study. The reduced longevity and very low fecundity of *E. connexa* on *T. ogmophallos* could be possibly due to lack of some nutrients essential for full longevity and good reproductive performance. According to Adams^59^ and Lima et al.^60^, adult females need suitable sources of nutrients to develop mature ovaries and produce eggs. Given that the period of the first oviposition (APOP and TPOP) was substantially longer for *E. connexa* on *T. ogmophallos*, it may be that the insufficient source of nutrients in this prey prolonged maturation of the predator’s ovaries before the first oviposition. The results are in compliance with those by Omkar and James^47^ and Tian et al.^25^ who report that prey suitability affects the oviposition of *Coccinella transversalis* Fabricius, *Coccinella undecimpunctata* (Linnaeus), and *Serangium japonicum* Chapin (Coleoptera: Coccinellidae). Furthermore, females that reached reproductive maturity by feeding on low-quality diet and acquired low energy during their development may be able to control the sex ratio of their offspring^61^. This would justify the male biased sex ratio (0.46) of *E. connexa* on *T. ogmophallos*, while the ratio is 1:1 (0.52) on *T. urticae* and *M. persicae.* Thus rearing of *E. connexa* on *T. ogmophallos* could lead to male individuals in the population, in response to the poor quality of the prey species and as a strategy to reproduce. However, further investigations should be carried out to demonstrate the likely impacts of these lower quality prey species on the predator.

The intrinsic rate of increase (*r*) of *E. connexa,* reflecting the effects of biological parameters such as development, survival, fecundity, and sex ratio^50^ on the predator’s population, was higher on *T. urticae* and *M. persicae* (0.118–0.126 day^−1^) compared to that on *T. ogmophallos* (0.047 day^−1^). Zazycki et al.^31^ under similar conditions reported a *r* comparable value (0.126 day^−1^) on pollen and *E. kuehniella* eggs for the aforementioned prey species.

Ocerall, these findings suggest that *T. urticae* and *M. persicae* are more suitable prey species for *E. connexa* than *T. ogmophallos,* while *T. evansi* is not suitable for this predator. Although enlightening, these results are certainly preliminary for the actual potential of *E. connexa* as biological control agent of spider mites and *M. persicae* in the field. Given that under natural conditions *E. connexa* is a generalist predator and can consume alternative as well as essential prey items, the performance of *E. connexa* could improve even with low-quality prey. The next steps should include predation capacity evaluation, weight and size of different life stages as well as field studies in order to determine the performance of this predator for the control of spider mites and *M. persicae* in the agroecosystems.

## Supplementary Information

Supplementary Figure 1.
