# Supplementary figures and images for: Suitability of spider mites and green peach aphids as prey for Eriopis connexa (Germar) (Coleoptera: Coccinellidae)
Source: Sci Rep. 2022 May 16;12:8029. doi: 10.1038/s41598-022-12078-8 (PMC9110729; doi:10.1038/s41598-022-12078-8)

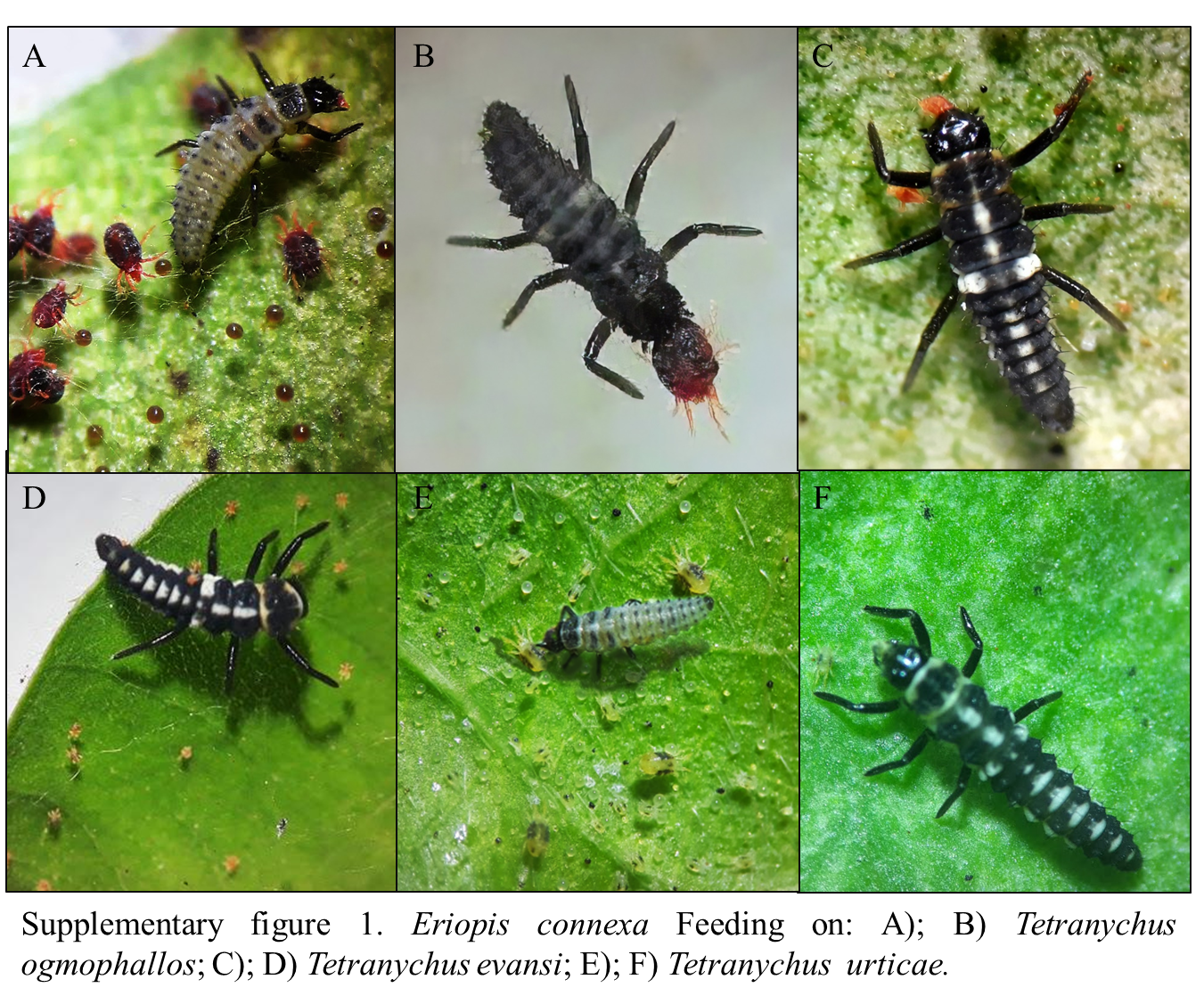

Supplement: Supplementary file 1 — Supplementary Figure 1. [file 41598_2022_12078_MOESM1_ESM.tif]
